# Perceptions and Attitudes Toward a Mobile Phone App for Mental Health for College Students: Qualitative Focus Group Study

**DOI:** 10.2196/18347

**Published:** 2020-08-07

**Authors:** Bree E Holtz, Alexis M McCarroll, Katharine M Mitchell

**Affiliations:** 1 Michigan State University East Lansing, MI United States

**Keywords:** mental health, mobile phone, mHealth

## Abstract

**Background:**

Many college students who have mental health issues do not receive professional care for various reasons. Students who do not receive help often have both short- and long-term adverse health outcomes. Mobile apps for mental health services such as MySSP, a service provided to college students through their university, may help eliminate barriers to seeking mental health care and result in more positive outcomes for college students.

**Objective:**

This qualitative study aims to better understand college students’ perceptions and attitudes toward the adoption and use of a mobile phone app for mental health, MySSP, using the technology acceptance model (TAM).

**Methods:**

A series of nine focus groups were conducted with college students (N=30) between February and May 2019 at a large, public Midwestern university. The moderator’s guide was based on the TAM, and focus group sessions primarily focused on the use and knowledge of apps for mental health, specifically, MySSP. The focus group transcriptions were hand-coded to develop a set of themes that encompassed students’ perceptions and attitudes toward MySSP.

**Results:**

The analysis of the focus groups suggested the following themes: (1) existing awareness of the app, (2) perceived usefulness, (3) perceived ease of use, (4) attitudes toward apps for mental health and MySSP, and (5) social influence.

**Conclusions:**

The results of this study provide deeper insights into the perceptions of a mobile app for mental health among college students. Future research should explore the specific contexts in which an app for mental health will be most effective for college students.

## Introduction

### Background

The prevalence and severity of mental health disorders among college students has been steadily rising [1]. Data from the American College Health Association showed that 45.1% of college students reported feeling so depressed that it was difficult to function and 65.7% felt overwhelmed with anxiety over the last 12 months [2]. In addition, from 2015 to 2019, there has been a 4% (from 9.3% to 13.3%) increase in students reporting that they have seriously considered suicide in the last 12 months [2,3]. However, the same report showed that only 24.3% of students had sought help for anxiety over the past 12 months and even fewer had sought help for depression (20%). Many college students who have mental health issues do not receive professional help because of the limited resources on college campuses, the time required to receive help, the lack of awareness of college mental health resources, and the stigma around receiving care [4-6]. Students who do not receive help have a higher incidence of dropping out before completing a degree [7,8] and experience long-term adverse outcomes, including low employment [9], perpetual emotional and physical health problems [10], and relationship dysfunction [11]. Consequently, many colleges and universities are seeking innovative ways to help students receive the help they need.

Mobile phones may prove to be a beneficial tool for providing mental health access and to overcome the barriers associated with receiving treatment. Mobile phones are ubiquitous among college students, with 95% of college students owning a smartphone [12]. College students and young adults are rarely without their phones, using them for several facets of their lives, generally through mobile apps. Some research suggests that college students would rather use in-person resources than web-based resources for mental health; however, college students are likely to use web-based resources because of their availability, convenience, and confidentiality [13-15]. Mobile apps for mental health services can provide users with additional benefits that seeking face-to-face help does not provide, such as its relatively low cost for care and the lack of stigma from seeking in-person treatment. In addition, mental health apps could be a helpful supplement to in-person care or as a first step in seeking in-person care.

Although there has been much research on mental health apps for college students, this qualitative study uses the technology acceptance model (TAM) as a framework to better understand college students’ perceptions and attitudes toward the adoption and use of a mobile phone app, MySSP (Morneau Shepell), for mental health [16,17]. MySSP is a mobile app service purchased by universities and is a resource for college students to receive mental health help, including support from professional counselors. As MySSP is readily available to participants through their university, this study focuses on perceptions and attitudes toward the adoption of this app specifically. Focus groups were conducted with both undergraduate and graduate students to explore their perceptions and use of mental health apps, specifically MySSP. This study can also provide insights into this population’s attitude of other apps that are developed to support mental health. In the next section *Literature Review*, we review the research related to mental health services via information and communications technologies (ICTs), including mobile phone apps for mental health for college students, followed by a brief review of the TAM. Finally, our research questions are presented.

### Literature Review

#### Mobile Health for Mental Health

There has been an increasing number of ICTs that seek to help people with mental health issues, such as web-based psychological therapy, remote video consultation, and social media platforms. The use of these technologies “represent a cultural change in mental health care by empowering patients to exercise greater choice and control” [18]. These apps aim to help with a variety of mental health conditions such as anxiety, depression, and obsessive-compulsive disorder, to name a few. Many of these apps attempt to provide different functionalities, such as controlled breathing, positive thinking, and meditation [19,20]. A vast majority of these apps remain unstudied and have not been thoroughly tested, leaving little evidence for their proposed benefits [20-22]. However, a handful of apps that have been studied provide valuable insights into their usefulness. Clinical trials that have been conducted sought to test the effectiveness of mobile apps for mental health, and the findings suggest that the apps are more beneficial for those with low or moderate levels of depression but can improve symptoms of depression and anxiety [23-25]. Further research has demonstrated that many of these apps had limited and short-term use because of some acceptance barriers; however, the results suggest that some treatment is better than no treatment [22,26-29].

#### Mental Health Apps and College Students

When examining college students’ openness and attitudes toward using mental health apps in a college setting, researchers found that 26.1% of college students were open to using mental health apps; however, only 7.3% had ever used such an app before [13]. The relatively low rate of mental health app adoption was attributed to participants having no current mental health needs, perceptions that mental health apps felt too impersonal, confidentiality concerns, and the utility of the apps [13,30]. Similar to the general population, college students have also tended to use mobile apps for mental health for short periods (≤4 weeks), although it is unknown if this short-term adoption can be attributed to feeling better, poor app design, student workload, or something else [13,14].

#### TAM

The TAM is one of the most widely used frameworks to examine the adoption of technology in a multitude of settings [16]. An extension of the theory of reasoned action [31], the TAM suggests that there are 2 key factors that predict the acceptance and use of a new technology, including perceived usefulness and perceived ease of use, both of which impact the attitude toward using a technology. Perceived usefulness is the users’ perceptions of how well a technology will improve their current practices. The ease of use is defined as how easy the technology is to learn and use. Attitude refers to the general feeling of a user when implementing a technology into their everyday routines. The TAM has been used in a variety of health contexts, including among college students and in mental health mobile apps [32]. Furthermore, the TAM has been used in the analysis of qualitative data using a grounded theory approach [33], but the TAM has also been used to deductively inform the development of semistructured interview protocols [34,35].

In addition, social factors deeply affect an individual’s attitude, and a multitude of theories posit that social influence is a key consideration in understanding adoption behaviors [31,36,37]. In the context of technology, the perception that the technology is accepted by one’s peers has been demonstrated to be an important factor of adoption [38]. Therefore, the individual’s attitudes, informed partially by their peers and partially by their evaluation of the technology (ie, ease of use and usefulness), will influence their overall intentions of acceptance of an app for mental health issues [39]. As college-aged adults have a greater communication with their peers about topics of personal importance such as health [38], we believe that social influence will be a salient factor for our population. Therefore, this study uses the TAM to deductively explore college students’ perspectives of perceived usefulness and ease of use, related to MySSP, which are key to understanding their intentions to use the app.

#### MySSP App Description

**Figure 1:**
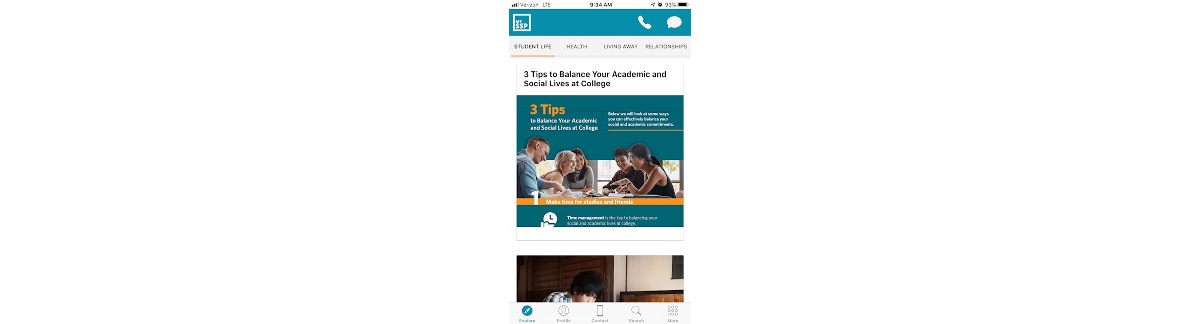
Student life screen of the MySSP app.

**Figure 2:**
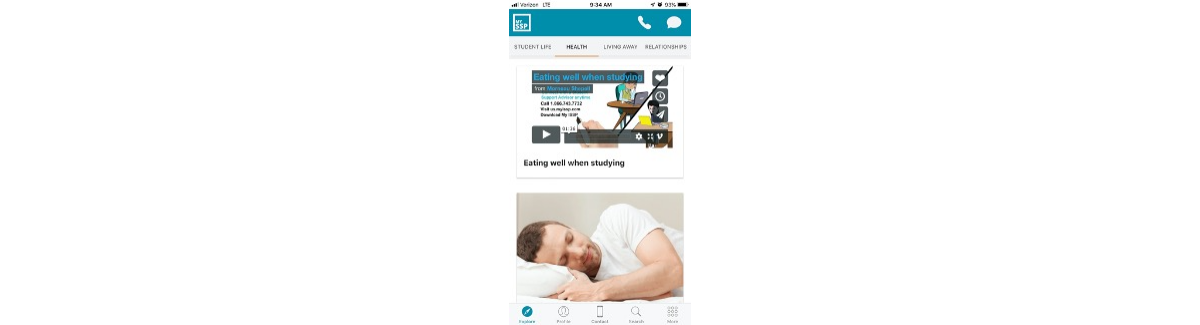
Health screen of the MySSP app.

**Figure 3:**
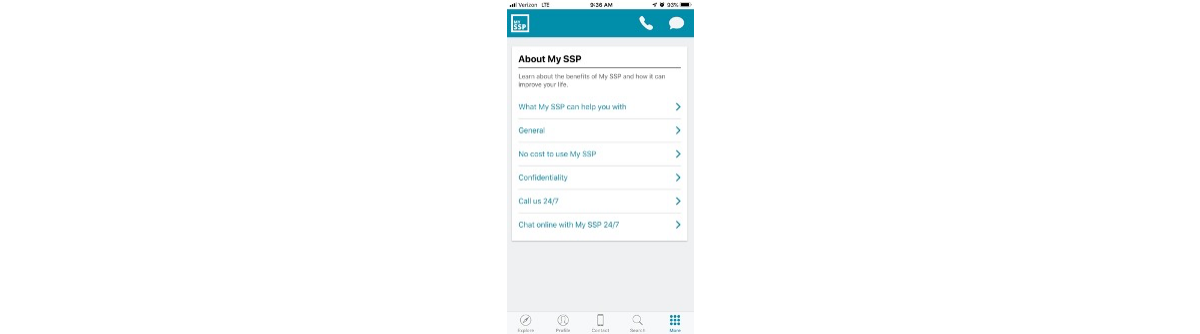
About screen for the MySSP app.

MySSP is a mental health services app, developed by Morneau Shepell, which is purchased by universities for their students. In the summer of 2018, the student health system of the university where this study took place made a contract with the company to provide mental health services to the university’s students. Once a contract is signed, the app is freely available to the university’s students providing immediate and confidential support from professional counselors through Morneau Shepell through chat (texting), voice, or video. The counselors are trained about the university and are connected to the university’s mental health staff. The app also provides text tips about mental health and well-being and provides a variety of informational articles that are relevant to the mental health and well-being of college students (eg, roommate problems, homesickness, stress around exams, etc). The app is available on the Google Play store and the Apple App Store. Figures 1-3 show app screenshots.

To promote the use of the MySSP app, the university sent out all-campus emails, promoted the service through several social media channels throughout the different departments and colleges, and posted flyers throughout the campus. Faculty members and academic advisors were given information about the resources and asked to share it with their students. In addition, students were told about the app if they called the campus counseling center.

This qualitative study aimed to further our understanding of college students’ attitudes and perceptions of mental health apps, specifically MySSP. The TAM guided the framework of the focus groups to better understand students’ intention to use the app. In addition, this formative research is a necessary first step in understanding how a campaign could be developed to further promote mental health awareness of campus resources. The research questions that guided this work included understanding the students’ general awareness of apps for mental health, specifically, the MySSP app. Once the MySSP app was demonstrated, we sought to understand their overall perceptions of the app.

## Methods

### Data Source and Participants

A series of 9 focus groups were conducted with college students (N=30) at a large, public, Midwestern land-grant university. The focus groups took place between February and May 2019. To be eligible, participants had to be currently enrolled at the university and be aged older than 18 years. The university’s institutional review board approved all portions of this study.

### Participant Demographics

Most participants were aged between 18 and 22 years (27/30, 90%) and were identified as female (25/30, 83%). Most participants were undergraduates, and seniors (fourth and fifth year) made up 27% (8/30) of the participants. Most participants indicated their race as white (19/30, 63%), followed by Asian or Pacific Islander (9/30, 30%). Participants were also asked in the demographic survey if they had ever used mental health services; the majority (16/30, 55%) stated that they had never used mental health services. They were also asked if they had used mental health resources in the last 12 months, and 7 students reported that they had used mental health resources. Table 1 provides participant demographics.

**Table 1:** Participant demographics (N=30).

Variables		Frequency, n (%)
**Age (years)**		
	18	1 (3)
	19	9 (30)
	20	6 (20)
	21	7 (23)
	22	4 (13)
	30	1 (3)
	33	1 (3)
	41	1 (3)
**Year in school**		
	First-year undergraduate	4 (13)
	Second-year undergraduate	7 (23)
	Third-year undergraduate	7 (23)
	Fourth-year undergraduate	8 (27)
	Graduate or professional student	4 (13)
**Gender**		
	Male	5 (17)
	Female	25 (83)
**Ethnicity origin (or race)**		
	White	19 (63)
	Black	1 (3)
	Asian or Pacific Islander	9 (30)
	American Indian	1 (3)
**Grade point average**		
	4.0	6 (20)
	3.5	14 (48)
	3.0	9 (31)
**Use of mental health services**		
	Yes	13 (45)
	No	16 (55)
**Use of mental health services (last 12 months)**		
	Yes	7 (24)
	No	22 (76)

### Procedure

Participants were recruited through the communication college’s SONA system, a subject pool software. The participants were paid $15 to complete the focus group. Participants sat around a table equipped with a recording device, and there was a notetaker present. Sessions were audiotaped and lasted approximately 60 to 90 min. The moderator’s guide was developed based on the TAM and included questions such as “Do you think that this app could provide mental health help/care?” and “What must this app have to be considered useful?” The session began with a short written demographic survey. Then, the focus group started with a brief discussion of general app use and was followed by a discussion of apps used for general health. This was done as an icebreaker for the sessions and to understand how general app use is similar to and different from apps for mental health. The majority of the session focused on apps used specifically for mental health, including their use and knowledge of the MySSP app. Following the discussion of apps for mental health, the moderator provided a brief overview of the MySSP app whereas the participants were given flyers, the informational email that had been sent to all students, and several screenshots of the MySSP app to review. After approximately 3 to 5 min of reviewing the materials, the participants were asked about their perceptions of usefulness, ease of use, and attitudes toward the MySSP app.

### Analysis

After each focus group, the notetaker and the moderator debriefed to develop a sense of several overarching themes. Data were analyzed using a descriptive analysis approach [40]. First, after the recordings of the focus groups were transcribed verbatim and returned to the research team, 3 members of the research team familiarized themselves with the data, reading, rereading, and making notes with initial ideas. This led the team to generate initial codes. We then iteratively developed a set of themes that captured the focus group dialog [41]. After the ninth focus group, the team concluded that saturation was reached. The themes were then presented to the whole team for clarification and feedback. Then, 2 members of the team coded a random selection (10%) of the transcripts to ensure intercoder reliability until reliability was reached (Cohen κ>0.8). Then, each coder independently coded half of the transcripts. Disagreements were identified and resolved by primary researchers. As a result of using the TAM to design the focus group protocol, the themes that emerged from the data were aligned with the TAM constructs. However, additional themes emerged from the analysis and are discussed in the Results section.

## Results

The results are organized based on themes that emerged from the descriptive analysis of the focus group, which included the constructs of the TAM and the additions of awareness of the app and social influence.

### Existing Awareness of the App

Although the app has been available to all students of the university and was announced through emails and flyers around campus starting in the fall of 2018 and continued through the spring of 2019 (when this study took place), very few students had even heard of the MySSP app. Of 30 participants, 7 (23%) had downloaded the MySSP app before attending the focus group. The remaining 23 participants (77%) had never heard of the MySSP app. During the focus group sessions, participants shared similar feedback about MySSP regardless of whether or not they had previously used the app.

### Perceived Usefulness

Most participants perceived that the MySSP app would be a useful tool for themselves, their friends, and other students at the university. Participants noted that MySSP would be useful for themselves, particularly in situations related to academic stress. For example, one participant said:

I know my friends, none of them have the same major as me so they don't really get my stress that I get from my classes. So maybe in that case if...a student was very stressed out, they could go talk to somebody about it

in the app

Another participant felt that MySSP would be useful among their friend group, particularly the instant messaging feature that allows users to *text* a counselor. The participant stated:

I like that you can text because a lot of my friends...we’re especially anxious about phone calls... texting is kind of stress free for us.

The participants also described several situations in which the MySSP app would be useful for other students at the university. One participant commented that the app would be particularly helpful to new students who may be having trouble adjusting to living in a new place. The participant said:

I think it would be a good resource for freshmen, especially if you're from out of state or you're [an] international [student] and you don't really know anybody. Or if you're having problems making friends, if you're stressed out, or homesick.

Participants also described scenarios in which a college student might need help navigating. For example, one participant said:

I feel like [MySSP] would also work out for people who are having relationship struggles...Maybe someone had a dramatic event happen...maybe they got a little too drunk at a party and they are trying to figure out and recollect what happened...

There was, however, some skepticism regarding the long-term usefulness of MySSP. One participant felt that the app was not equipped to deal with more serious mental health issues, saying:

I feel like it can help in maybe a crisis or if you're feeling lost, but I don't [think]...in the long term, it can do a ton...I think this is way too general for if someone has clinical depression, anxiety, specific OCD...it's more for people who are realizing that they might need help.

Another participant felt that MySSP would not have the same impact as in-person care in the long run but would still be useful in certain scenarios. This participant stated:

I think this mostly seems to be a gateway to something, I don't think it's going to be the same as a therapist or anything but for a lot of problems that students face around here like stress, breakups, relationship issues...this would really work well.

### Perceived Ease of Use

In general, health app use and retention varied among participants. Much of the feedback around short-term use is related to a lack of ease of use. For example, one participant noted how an app they used was too tedious to keep up with, saying:

I have this app downloaded but I haven't been keeping up with it...it's just really tedious because you have to really say how has your day been...you have to get all introspective.

Other participants mentioned that their short-term use was because they were focused on a certain goal (ie, losing weight), and once they achieved the goal, they no longer found a need for that particular app.

After the MySSP app presentation, all participants perceived the app to be easy to use. One participant specifically mentioned the ease of communicating:

I think it sounds like it’s quite easy...to get in touch with someone and there’s a lot of different methods of communication you can use if phone calling is just not your thing, for example.

Another participant echoed that perspective, saying:

I’d say that it’s free to all students...and that it’s anytime, anywhere that you can be in contact with someone [a counselor], so accessible.

In addition, participants appreciated that the app was available in multiple languages. One participant said:

I like the fact it says you can access an advisor or counselor who speaks your preferred language and understands your culture. That’s helpful for an international student.

### Attitudes Toward Apps for Mental Health and MySSP

As expected, the majority of the student participants used social media apps such as Facebook, Instagram, and Snapchat the most. In addition, many participants had experience using general health apps. However, very few of the participants used or had ever used an app for mental health. For example, one participant reported:

I haven’t really tried them [mental health apps]. I don’t know, sometimes my phone stresses me out so I don’t want to be on that to try to relax.

Many of the participants perceived that the app could be beneficial. For example, one participant appreciated that MySSP could provide her quick access to care, saying:

I’ve had interest in going out to find a therapist...but I feel like because we’re all so busy all the time it’s hard to go out of your way to just talk to somebody. I feel like having access through your phone is something that’s really cool.

Another participant noted that as she would not personally use MySSP for *her* mental health, others in more serious situations might use MySSP. She went on to say:

I feel like it just depends where someone is at the stage in their mental health...I wouldn’t use it for my mental health. But then other people may be need it in a crisis or just having that person to talk to, so I feel it just depends or where you’re at or what you need.

Some participants felt that MySSP could address a spectrum of mental health needs. One participant said:

I feel like every student could benefit from it. People that maybe have several problems, or somebody that is just having a bad day and needs somebody to talk to.

Participants also approved the appearance of the app, which impacted their perception of the quality of care. Regarding the quality, one participant shared:

I’d say it looks a lot better than I had imagined it. So, I think it looks very professional. So, I think the quality of help you would be getting would be more professional

as well

Overall, the majority of the participants had positive feelings about the app and that the university was looking for different ways to help service students’ mental health issues.

However, some of the participants did not believe that an app was the best way to receive mental health services. For example, a handful of participants felt that they would turn to family or friends for mental health support before turning to an app. One participant said:

I can’t really imagine that, because I think whatever problems I have, I can talk to my boyfriend first, or my sister, and also my friends.

Some other participants felt that they did not have any mental health concerns that were *serious* enough to justify using a mental health app. When asked about their attitudes toward using a mental health app for themselves, one participant shared:

I just feel that I don’t really need to [use a mental health app], I’m not in the state where I [would] download an app to take care of [my mental health]. Most of it is just like situational stress with test or quizzes or things like that. It’s not reoccurring or continuing.

Another participant expressed a similar sentiment saying:

I don’t have [a] mental health disorder, but everyone gets stressed and I like the way most people cope with it is trying to take their mind off of it. For me, that’s just listening to music or something. So, I don’t need to go to the extent of doing that

using a mental health app

### Social Influence

Participants were asked if they had ever downloaded an app because a friend or family member recommended it. Many participants had downloaded an app based on others’ recommendations. One participant said:

If I’m talking about something interesting and they mention something [about an app], I don’t really take a second thought, I’ll say, “Oh yeah that sounds good.” I’ll add it to my phone without thinking about it.

However, the apps that were recommended to participants were not mental health apps, rather games, photo editing, music streaming, or networking apps.

Researchers asked participants if they thought their friends would use the MySSP app. The majority of participants thought that if their friend needed mental health help and was aware of MySSP, then they would use it. One participant reiterated that MySSP is particularly useful in that their friend would be more likely to use the app than see a counselor face-to-face, saying:

I know my one friend struggled with depression and she’s always just like, she wants help, but she doesn’t want to walk. She’s like I don’t have time to walk to this place, or whatever, but since that’s available 24/7, and it’s just on your phone. You always have your phone on you, that would be really good.

Participants also mentioned their thoughts if they found that their good friend was using MySSP. Some responses included “Good that they’re getting help” and “I’d feel happy for them that they were doing something about the problem they had.”

## Discussion

### Principal Findings

Using the TAM, this study was an initial exploration of college students’ perceptions, attitudes, and intentions of using a mobile app for mental health. The results indicate that most students were not aware that MySSP is an available campus resource for mental health issues. Although the app was perceived by the participants, on initial judgment, it was useful and easy to use. Overall, they had positive attitudes toward the app; however, most participants did not perceive that they needed MySSP because they did not have any mental health issues. In addition, social influence appears to be a key component in college students’ use of apps. This study furthered our understanding of how TAM can be applied in a university setting for perceptions of mobile health for mental health, using a descriptive analysis to explain reasons for app adoption for mental health by college students.

As the results suggest, students were not aware of the app, which hinders the use and adoption of any intervention, not just this particular one. It is key that when an organization rolls out a resource, it effectively promotes the resource and finds champions who can share the information [42]. Past research shows that the students most likely to use these types of apps are generally women and those with lower levels of depression and anxiety [23,41]. However, male college students also face elevated rates of anxiety and depression. Feedback from the participants will be used to help inform the university on how to better promote mental health resources on campus. In addition, future research should consider how to connect with students who may need the most help.

The multiple modes available for communicating with a counselor, particularly through texting, were seen as key usefulness attributes of MySSP. Physically attending mental health counseling is often time consuming and can leave the student feeling stigmatized [43]. The more anonymous nature of the mediated communication via the app appeared to increase the perceived usefulness for many of the college student participants. In addition, communication mediated through an app is likely to increase rates of self-disclosure, which may positively impact long-term mental health outcomes [44]. Conversely, and similar to past research, participants indicated that the app may not be effective for longer-term use but rather as a tool to overcome the barriers to initial face-to-face counseling. The specific context in which an app for mental health is used is essential to college students’ perceived usefulness. Further research should consider the context in which an app, such as MySSP, is most useful for students seeking mental health help.

Although some mental health apps offer a wider variety of features to users, this can increase the complexity of the app. The TAM suggests that apps that are perceived to be easier to use are more likely to be accepted by users [45]. Previous research has found that perceived usefulness and perceived ease of use affect young people’s health app acceptance and effectiveness [17]. Participants noted that MySSP appeared to be easy, understandable, and accessible, which are all indications of an overall positive perception of ease of use. Goal setting was mentioned as a context in which long-term use may not apply; however, general health literature suggests that small, specific, and sustainable goals set and achieved over a period of time lead to positive long-term changes [46]. Therefore, with the help of a counselor, the app could implement goal setting in an effective way that actually increases use.

Many participants indicated that they did not feel they need an app for mental health, although the rates of college students who indicate facing issues of mental health are at historic highs [2]. The majority of college students reported feeling stressed on a regular basis. Previous research has indicated that this reoccurring stress can lead to anxiety and depression, in which college students are particularly susceptible. Furthermore, there appears to be a norm in which this stress, anxiety, and depression are accepted by college students. It is possible that the largest barrier to seeking help for mental health, whether face-to-face or via a mobile app, is because of the normative nature of these mental health issues during college. In addition, it is critical to note that when the students were discussing that they would likely not use MySSP, many of the comments were related to feeling like they did not need any professional mental health resources in any form. However, the students in this study did indicate that MySSP would likely help other students overcome the barriers frequently cited for not receiving face-to-face help. This indicates that factors other than barriers to seeking mental health help should be considered in future research.

Social influence is another key factor in establishing intention to use and the effectiveness of health apps [17]. The participants recalled many instances in which they downloaded an app because a friend or family member recommended it. Although none of the participants had experience with a mental health app being recommended to them, the power of peer influence in the uptake of mental health apps may be an avenue for increased use. In addition, participants reflected on how they would feel if their friend used an app for mental health. This feedback further confirmed that college students could benefit from an app for mental health and that the social attitudes around the use of mental health resources are generally positive.

### Limitations

As with most research, this study also has some limitations. First, the population of the focus groups was mostly white women, which does not reflect the university’s overall population. In addition, all of the participants had a grade point average of 3.0 or higher, indicating that they may be more highly motivated than the general student body. This is also indicated by their willingness to participate in the focus group. The students were also recruited through a communication college SONA system, which may not be representative of the student body. In addition, because mental health issues are often stigmatized, we might not have been able to get everyone’s true opinions regarding the issue. However, the potential use of the MySSP app was able to draw out more neutral experiences. Therefore, these results are still useful when developing a campaign promoting mental health resources, especially at larger public universities.

Our formative research into students’ perceptions of app use for mental health provides a path to further explore this issue. A strength of this study is that there were screenshots of an existing app for students to look at, and they could download the app to use during the session. In addition, some students in the focus groups had prior experience using the app. This provided concrete examples rather than an *idea* or concept of what a mobile app for mental health might look like. Using our findings, we have developed and are currently surveying the population about their mental health and intentions of using this app as a resource. This should provide us with more rigorous data and additional results.

### Conclusions

This study provides a deeper understanding of the perceptions of college students regarding a mobile app for mental health. The feedback from students will help the student health center promote the university’s resources for mental health help and encourage their use. Although this study used the TAM to understand students’ perceptions and attitudes toward a mental health app, further research is needed into the specific contexts in which an app for mental health will be most effective for college students. Furthermore, future work must identify why there is a gap between rates of depression and anxiety among college students and their intention to use mental health services.

## Abbreviations

ICT: information and communications technology
TAM: technology acceptance model
